# Prognostic and immunotherapeutic significances of M2 macrophage-related genes signature in lung cancer

**DOI:** 10.7150/jca.98044

**Published:** 2024-07-22

**Authors:** Haixia Wu, Yilin Yu, Wei Wang, Gen Lin, Shaolin Lin, Jiguang Zhang, Zhaojun Yu, Jiewei Luo, Deju Ye, Wu Chi, Xing Lin

**Affiliations:** 1 Shengli Clinical Medical College of Fujian Medical University, Fuzhou, Fujian, China.; 2 Clinical Oncology School of Fujian Medical University, Fujian Cancer Hospital, Fuzhou, Fujian, China.; 3 Department of Thoracic Surgery, Fujian Provincial Hospital, Fuzhou, Fujian, China.; 4 Fujian Provincial Key Laboratory of Emergency Medicine, Fujian Provincial Institute of Emergency Medicine, Fujian Emergency Medical Center, Fuzhou, China.; 5 State Key Laboratory of Analytical Chemistry for Life Science, School of Chemistry and Chemical Engineering, Chemistry and Biomedicine Innovation Center (ChemBIC), Nanjing University, Nanjing, China.

**Keywords:** M2 macrophages, Lung cancer, M2 macrophage-related risk score, Tumor microenvironment, Immunotherapy response.

## Abstract

**Objective:** We aimed to investigate the immunological significance of M2 macrophage-related genes in lung cancer (LC) patients, specifically focusing on constructing a risk score to predict patient prognosis and response to immunotherapy.

**Methods:** We developed a novel risk score by identifying and incorporating 12 M2 macrophage-related genes. The risk score was calculated by multiplying the expression levels of risk genes by their respective coefficients. Through comprehensive enrichment analysis, we explored the potential functions distinguishing high- and low-risk groups. Moreover, we examined the relationship between patients in different risk groups and immune infiltration as well as their response to immunotherapy. The single-cell RNA sequencing data were acquired to ascertain the spatial pattern of RNF130 expression. The expression of RNF130 was examined using TCGA datasets and verified by HPA. The qRT-PCR was employed to examine RNF130 expression in LC cells. Finally, *in vitro* experiments were carried out to validate the expression and function of RNF130.

**Results:** Our results indicated that the risk score constructed from 12 M2 macrophage-related genes was an independent prognostic factor. Patients in the high-risk group had a significantly worse prognosis compared to those in the low-risk group. Functional enrichment analysis showed a significant relationship between the risk score and immunity. Furthermore, we explored immune infiltration in different risk groups using seven immune algorithms. The results demonstrated a negative correlation between high-risk group patients and immune infiltration of B cells, CD4+ cells, and CD8+ cells. We further validated these findings using an immunotherapy response database, which revealed that high-risk patients were more likely to exhibit immune evasion and might have poorer immunotherapy outcomes. Additionally, drug sensitivity analysis indicated that patients in the high-risk group were more sensitive to certain chemotherapeutic and targeted drugs than those in the low-risk group. Single-cell analysis indicated that macrophages were the primary site of RNF130 distribution. The results from the TCGA and HPA database demonstrated a trend toward a low expression of RNF130 in LC. Finally, *in vitro* experiments further validated the expression and function of RNF130 in LC cells.

**Conclusions:** The high-risk group constructed with M2 macrophage-related genes in LC was closely associated with poor prognosis, low immune cell infiltration, and poorer response to immunotherapy. This risk score can help differentiate and predict the prognosis and immune status of LC patients, thereby aiding in the development of precise and personalized immunotherapy strategies.

## Introduction

Lung cancer (LC) is the major cause of cancer death globally. According to the most recent data, it will claim the lives of over 350 people each day 1. Despite the availability of surgical intervention, chemotherapy, targeted therapy, and immunotherapy for LC patients, the overall survival (OS) rate remains significantly unsatisfactory 2. Among the various treatment options, immunotherapy is the most promising for patients with LC, revolutionizing anti-tumor therapy and ushering in a new era 3. However, there is still a need for greater clarity regarding the specific features of patient groups that would benefit from immunotherapy and the predictors associated with this benefit 4.

There is increasing evidence that macrophages play important roles as mediators coordinating the interaction between the immunological defense of tumors and the potential anti-tumor actions of the immune system 5. The macrophage phenotype is plastic in response to the microenvironment and signals, with two primary subsets: conventionally activated (M1) and alternatively activated (M2) macrophages^6^. In general, M1 macrophages secrete pro-inflammatory cytokines that contribute to the destruction of tumor cells, while M2 macrophages secrete anti-inflammatory cytokines that promote tumor angiogenesis and growth 7.

Most clinical researches have indicated that tumor-associated macrophages infiltration in solid tumors is correlated with the expression of genes associated with M2 gene profiles 8, 9. By secreting a variety of immunosuppressive cytokines, M2 macrophages weaken the immune system, thereby promoting tumor growth 10. M2-tumor associated macrophages constitute a significant group that impedes the activation and infiltration of CD8+ T lymphocytes in the tumor microenvironment 11. There is extensive evidence indicating that M2 macrophages play a significant role in tumor progression 12. Lan *et al.* found that exosomes generated from M2 macrophages enhance the migration and invasion of colon cancer cells 13. Inducing M2 polarization of macrophages in tumor microenvironments has been found to promote breast cancer progression 14 and increase pancreatic cancer metastasis 15. Additionally, Wei *et al.* reported a close correlation between M2 macrophage infiltration and LC prognosis 16. Consequently, investigating the fundamental function of M2 macrophages and their associated molecules in LC is imperative.

In this study, our aim was to investigate a predictive marker for LC using M2 macrophage-related genes. We obtained RNA-seq data from The Cancer Genome Atlas (TCGA) and the Gene Expression Omnibus (GEO) datasets. Then, we identified 12 genes by least absolute shrinkage and selection operator (LASSO) regression analysis. The risk score was calculated by multiplying the expression of risk genes by their respective coefficients. Patients were then categorized into high- and low-risk groups based on this score. Through gene set enrichment analysis (GSEA), we explored potential functional differences between high- and low-risk groups. Besides, we examined the relationship between different risk groups and immune infiltration, as well as their response to immunotherapy. We also investigated the differences in drug sensitivity among patients in different risk groups. The single-cell RNA sequencing data were acquired to ascertain the spatial pattern of RNF130 expression. The expression of RNF130 was examined using TCGA datasets and verified by the Human Protein Atlas (HPA). The quantitative real‑time polymerase chain reaction (qRT‑PCR) was employed to examine RNF130 expression in LC cells. Finally, *in vitro* experiments were carried out to validated the expression and function of RNF130. Our findings suggested that M2 macrophage-related genes may have a role in the prognosis of LC, offering insights into the function of these genes in the LC tumor microenvironment and identifying potential therapeutic and prognostic targets for LC.

## Methods

### Dataset acquisition and processing

We acquired expression profiles and clinical data of LC patients from two databases, namely the TCGA and GEO, which provide open access to this information. A comprehensive analysis was conducted on the combined datasets from the TCGA, GSE50081, GSE30219, GSE31210, and GSE37745 cohorts to enhance the robustness and generalizability of the findings. LC patients included in our study met the following criteria: (a) Confirmed diagnosis of LC based on histopathological examination; (b) Availability of gene expression data and corresponding clinical information; (c) Adequate follow-up information to assess clinical outcomes. Exclusion criteria: (a) Patients with missing or incomplete clinical data, including survival outcome or treatment information. (b) Patients with significant comorbidities that could confound the expression status of M2 macrophage-related genes and prognosis; (c) Patients with incomplete gene expression profiles or low-quality data in the TCGA and GEO databases; (d) Patients with insufficient follow-up duration to evaluate long-term survival outcomes. R and Perl scripts were used to analyze the raw data.

### Identification of M2 macrophage-related genes in LC

We obtained M2 macrophage-related genes from the TCGA database in LC patients. M2 macrophages abundance were estimated using CIBERSORT 17, and genes were selected based on a correlation greater than 0.3 and a p-value less than 0.001. Comprehensive networks were then employed to investigate the relationship between these genes and M2 macrophages.

### Biological enrichment analysis

The "ClusterProfiler" R package 18 was utilized to conduct Gene Ontology (GO) and Kyoto Encyclopedia of Genes and Genomes (KEGG) analysis. Statistical significance for both analyses was determined using a p-value threshold of less than 0.05.

### LASSO regularization and development of M2 macrophage-related risk score

Within the TCGA training cohort, univariate Cox regression analysis was conducted to explore the association between patient survival and the expression of risk genes. In order to further refine prognostic genes selection, LASSO cross-validation approaches were applied using the "glmnet" R package, with a significance threshold of p<0.05. The risk score was defined as the expression of genes multiplied by their respective coefficients. Based on the median value, patients were stratified into low- or high-risk group. To compare the two groups' survival rates, we utilized Kaplan-Meier survival analysis and a bilateral log-rank test to analyze differences. The R packages "Survcomp" and "SurvivalROC" were utilized to construct receiver operating characteristic (ROC) curves for assessing the predictive power of the risk score. Furthermore, we independently utilized three GEO databases to validate the prognostic significance of the risk score for LC patients. Finally, a nomogram was developed using clinical features and the risk score through the utilization of the "rms" package. Each variable in the nomogram scoring system was assigned a score, and the total score for each sample was calculated by summing all scores. The prognostic power was assessed using the testing sets from Datasets GSE50081.

### Gene set enrichment analysis of low-risk and high-risk groups

We used the GSEA approach to identify potential biological functions and pathways in the low- and high-risk groups. HALLMARK gene sets (c5.go.symbols.gmt and c2.cp.kegg.symbols.gmt) sourced from the MSigDB were utilized for analyses 19, 20. For each analysis, 1000 gene set permutations were performed. A normalized enrichment score (NES) greater than 1 or less than -1, and a false discovery rate (FDR) value less than 0.05, were deemed to indicate significant enrichment in each phenotype.

### Comparative analysis of immune infiltration between low-risk and high-risk groups

The CIBERSORT algorithm was used to evaluate the correlation between risk groups and immune cell infiltration 17. Besides, eight immune checkpoint-related genes were selected to assess their association with the risk score. Additionally, we employed six algorithms (xCell, EPIC, quantiseq, TIMER, MCPcounter, and ESTIMATE) to evaluate the immunological cell abundance across different risk categories 21-26. To determine the amount of immune infiltration in each sample, the “IOBR” function of the R package was utilized to compute the TCGA expression matrix for the training set, encompassing important immune-infiltrating cells such as CD4+ T cells, CD8+ T cells, macrophages, B cells, and NK cells.

### Evaluation of immunotherapy response

In order to investigate the relationship between the risk group and immunotherapy response, the Tumor Immune Dysfunction and Exclusion (TIDE) analysis tool (http://tide.dfci.harvard.edu/) was utilized 27, 28. LC patients were allocated a TIDE score according to their normalized expression profile. TIDE was used to obtain the TIDE, MSI Expr Sig, CD274, CD8, Dysfunction, Exclusion, MDSC, CAF, and TAM M2 treatment scores of each LC sample.

### Exploring drug sensitivity between low-risk and high-risk groups

Information on drug sensitivity was extracted from the Genomics of Drug Sensitivity in Cancer (GDSC) database 29. Predictions were made regarding drug sensitivity in patients between the two different risk groups. The half inhibitory concentration (IC50), representing the concentration of a drug inhibiting half of the maximum response, was selected as the benchmark for comparing pharmacological responses across different risk groups.

### Single cells analysis

The Tumor Immune Single Cell Center (TISCH) database, a repository of single-cell RNA data (http://tisch.comp-genomics.org), was utilized to evaluate the expression level of RNF130 in various cell types within the tumor microenvironment 30.

### HPA databases

The HPA provides a comprehensive map of all the proteins found in human cells, tissues, and systems (www.proteinatlas.org) 31, 32. The protein expression of RNF130 in normal and LC tissues was compared using the HPA.

### Cell lines culture

The Beas-2a, A549, H1299, PC9, HCC827, and H1975 cells were purchased from FuHeng Cell Center, Shanghai, China. The cells were grown in RPMI-1640 media containing 10% fetal bovine serum and 1% penicillin-streptomycin. The incubator was configured to maintain environmental conditions at a temperature of 37°C and a CO2 concentration of 5%.

### qRT‑PCR

The RNA was isolated using TRNzol Universal Reagent from Tiangen Biotech (China). Reverse transcription was carried out with Tiangen Biotech's FastKing gDNA Dispelling RT SuperMix. Applied Biosystems' StepOnePlus System was used for quantitative PCR with SuperReal PreMix Plus from Tiangen Biotech. The fold change of gene expression was calculated using the 2^-ΔΔCT^ method, with ACTIN serving as the normalization control. The primer sequences utilized in this work were included in **Supplementary Table s1**. The PCR reaction was conducted three times.

### Cell transfection

RNF130 silencing in A549 cells was synthesized by transfection with RNF130 siRNA (Sangon, China). The transfection was performed using lipofectamine™ 3000 (Thermo Fisher Scientific, USA) according to the manufacturer's instructions. The siRNA sequences were shown in **Supplementary Table s2**.

### Cell proliferation assays

The evaluation of cell proliferation was conducted using the cell counting kit-8 (CCK-8) assays (APExBIO, USA). At 24-, 48-, and 72-hours post-transfection, 10 μL of CCK-8 reagent was introduced to each well, and the absorbance was quantified at a wavelength of 450 nm by Multi-Mode Microplate Reader (SpectraMax ID5, USA). Each experiment was repeated three times.

### Wound healing assay

A549 cell transfected with si-RNF130 were seeded in 6-well plates. Once the cell density reached 90-100%, we utilized a 10 µl pipette tip to create a straight wound by scratching. Microscopic images were captured at 0 and 48 hours, and the experiment was replicated three times.

### Statistical analysis

The data were visualized and statistically analyzed using R version 4.3.1 and Perl version v5.30.0. Differences between groups were assessed by two tailed Student's t-tests, χ2 test, or Wilcox tests. Kaplan-Meier analysis and the Log-rank test were used to examine the survival data. To determine the correlation coefficients, Spearman's correlation analyses were utilized as the appropriate method. The risk score's validity was validated using the ROC curve. In this study, all tests were two-tailed, and statistical significance was defined as a p-value<0.05.

## Results

### Identification of genes associated with M2 macrophages in LC

**Figure 1:**
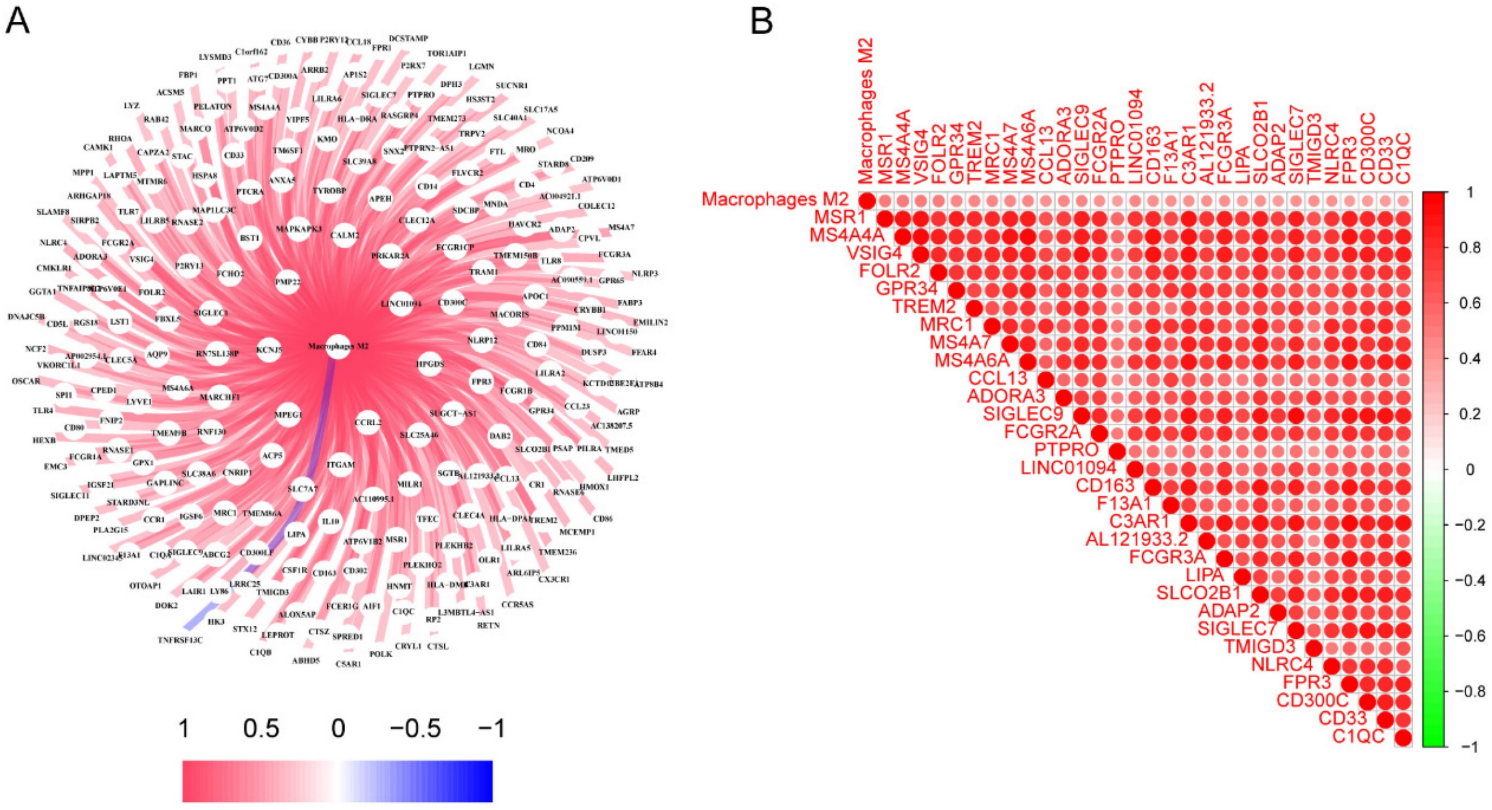
The identification of genes associated with M2 macrophages in LC. (A) Interconnected networks between genes related to M2 macrophages and M2 macrophages; (B) The correlation between the top 30 genes associated with M2 macrophages. LC, lung cancer.

The characteristics of the patients were presented in **Table 1**. A total of 245 genes associated to M2 macrophages were discovered. A thorough network analysis was presented to investigate the correlation between these genes and M2 macrophages (**Figure 1A**). In addition,** Figure 1B** showed the top 30 genes associated with M2 macrophages. Notably, each of these genes exhibited a positive association with M2 macrophages, and the top 30 genes also demonstrated a positive correlation with each other.

### Biological enrichment analysis of M2 macrophage-related genes in LC

**Figure 2:**
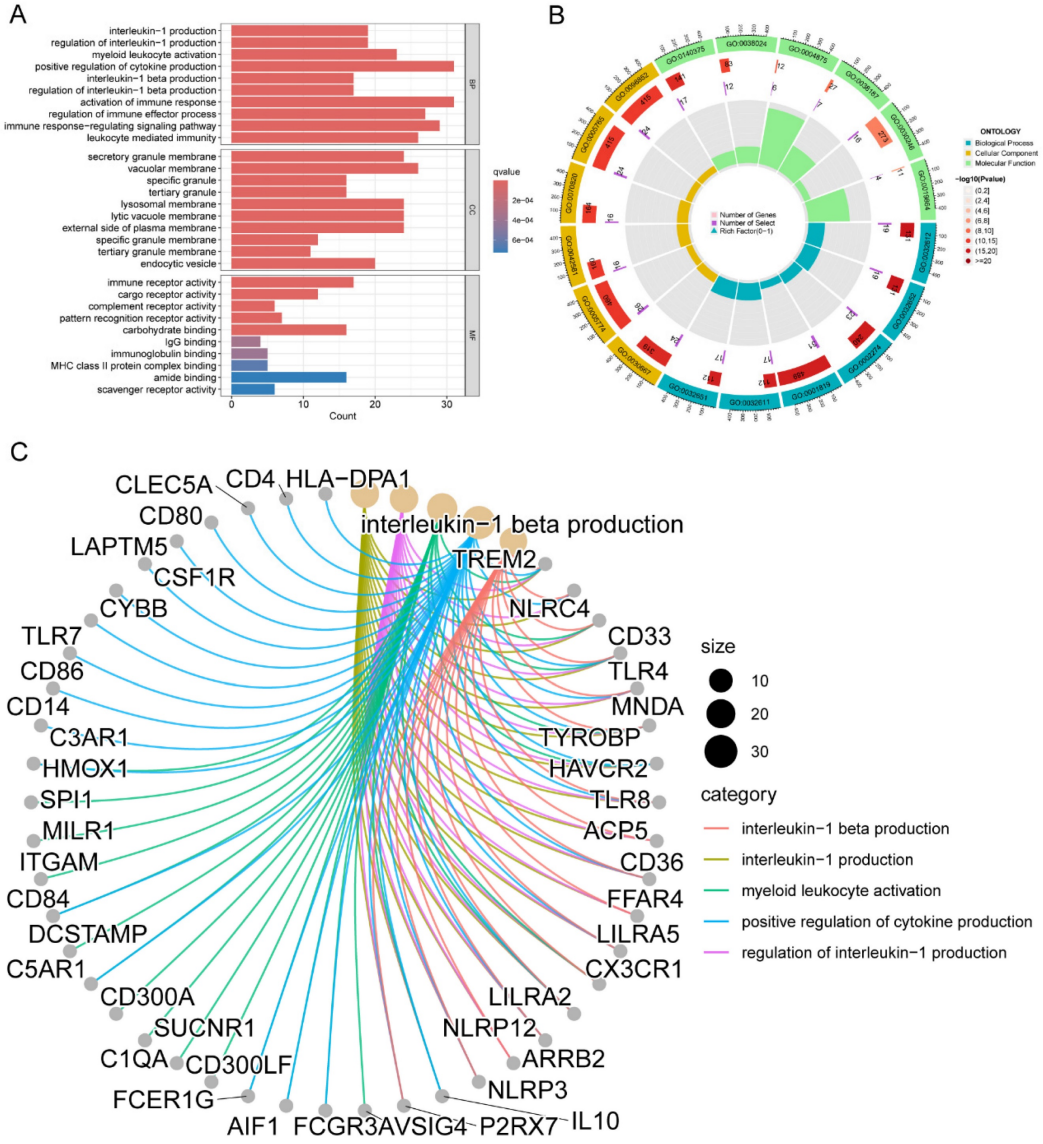
Functional enrichment analysis of genes associated with M2 macrophages in LC. (A) BP, CC, and MF of GO analysis for M2 macrophage-related genes; (B) BP, CC, and MF of the top six GO analysis for M2 macrophage-related genes; (C) Interconnected networks of GO BP and M2 macrophage-related genes. LC, lung cancer; BP, biological process; CC, cell component; MF, molecular function; GO, Gene Ontology.

Functional and pathway enrichment analysis were conducted to gain a deeper understanding of the potential role of M2 macrophage-related genes in LC. A total of 245 genes were investigated in order to identify 707 GO terms and 10 KEGG pathways. The following categories were defined and presented: biological processes (BP), cellular components (CC), and molecular function (MF) (**Figure 2A**). Terms such as interleukin-1 production, myeloid leukocyte activation, positive regulation of cytokine production, activation of immune response, regulation of immune effector process, immune response-regulating signaling pathway, and leukocyte mediated immunity were enriched in the BP category; secretory granule membrane, lysosomal membrane, and external side of plasma membrane were enriched in the CC category; immune receptor activity, IgG binding, immunoglobulin binding, and MHC class II protein complex binding were enriched in the MF category.

**Figure 3:**
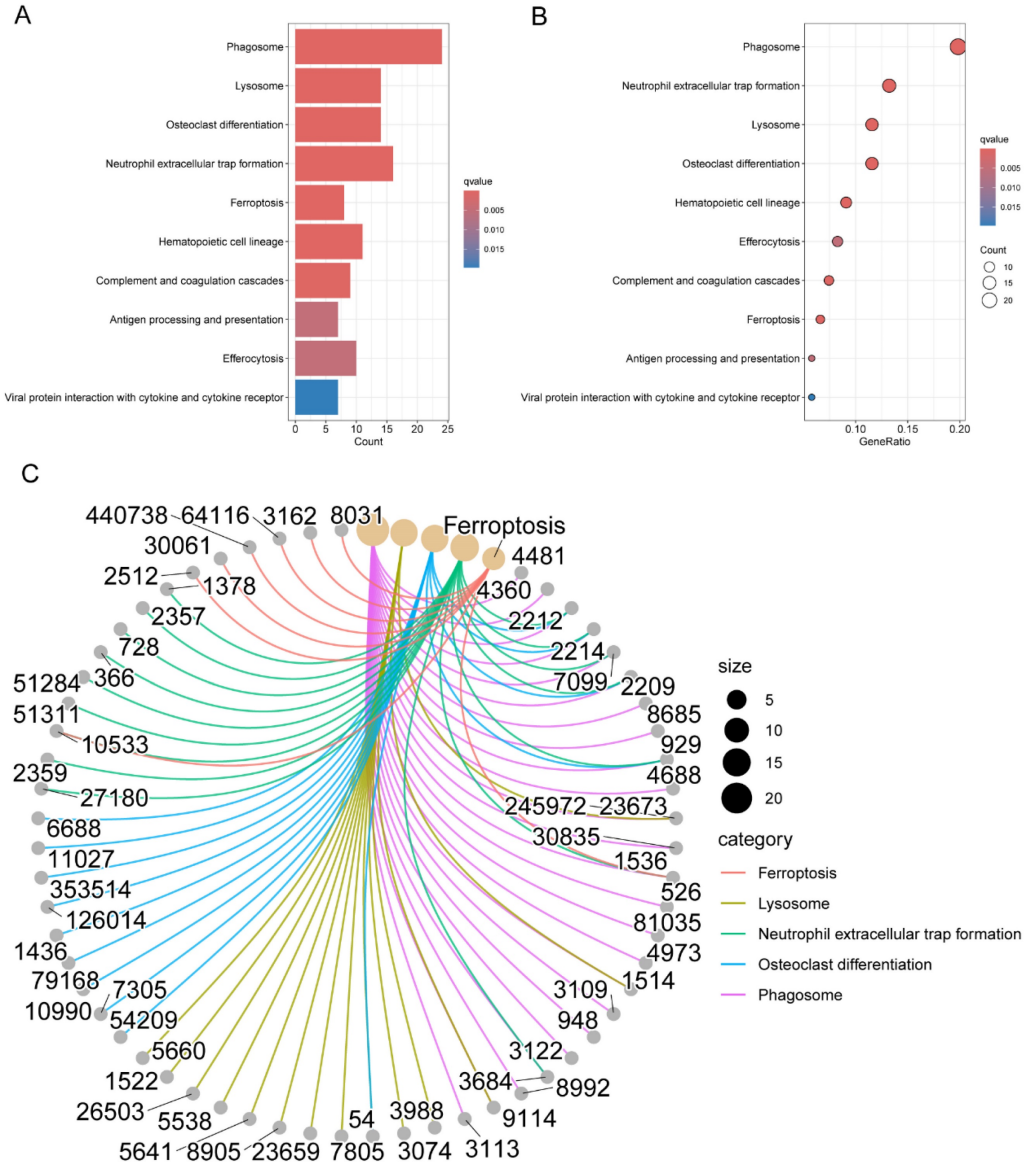
Pathway enrichment analysis of genes associated with M2 macrophages in LC. (A-B) KEGG pathway analysis for M2 macrophage-related genes; (C) Interconnected networks of KEGG pathways and M2 macrophage-related genes. LC, lung cancer; KEGG, Kyoto Encyclopedia of Genes and Genomes.

**Figure 2B** presented the top six enriched functional categories for BP, CC, and MF in a circular diagram. In addition, comprehensive networks were created to investigate the interaction between the genes connected to macrophages and the BPs of GO, as shown in **Figure 2C**. Regarding the KEGG pathways, M2 macrophage-related genes was primarily enriched in pathways related to phagosome, lysosome, neutrophil extracellular trap formation, antigen processing and presentation, and cytokine and cytokine receptor (**Figure 3A-C**). These findings demonstrated a strong correlation between M2 macrophage-related genes and the immunological response.

### Development of a M2 macrophage-related genes prognostic risk score

**Figure 4:**
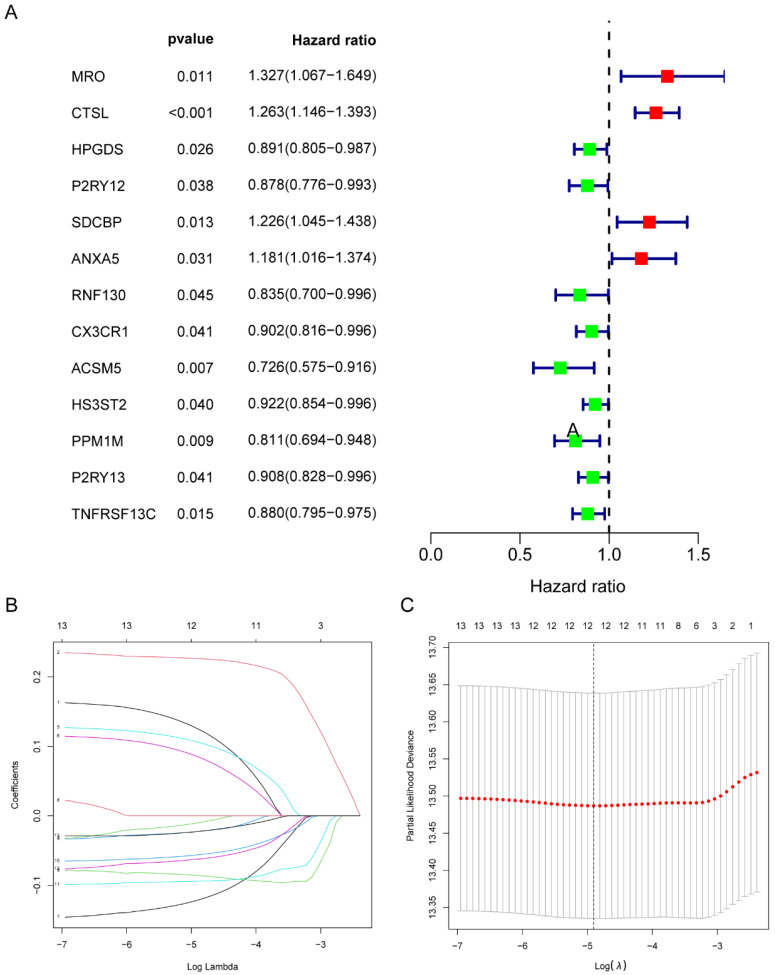
Identification of twelve M2 macrophage-related genes in LC. (A) Univariate Cox analysis of 13 M2 macrophage-related genes with prognostic value; (B-C) Cross-validation and LASSO regression analysis of M2 macrophage-related prognostic genes. LC, lung cancer.

**Figure 5:**
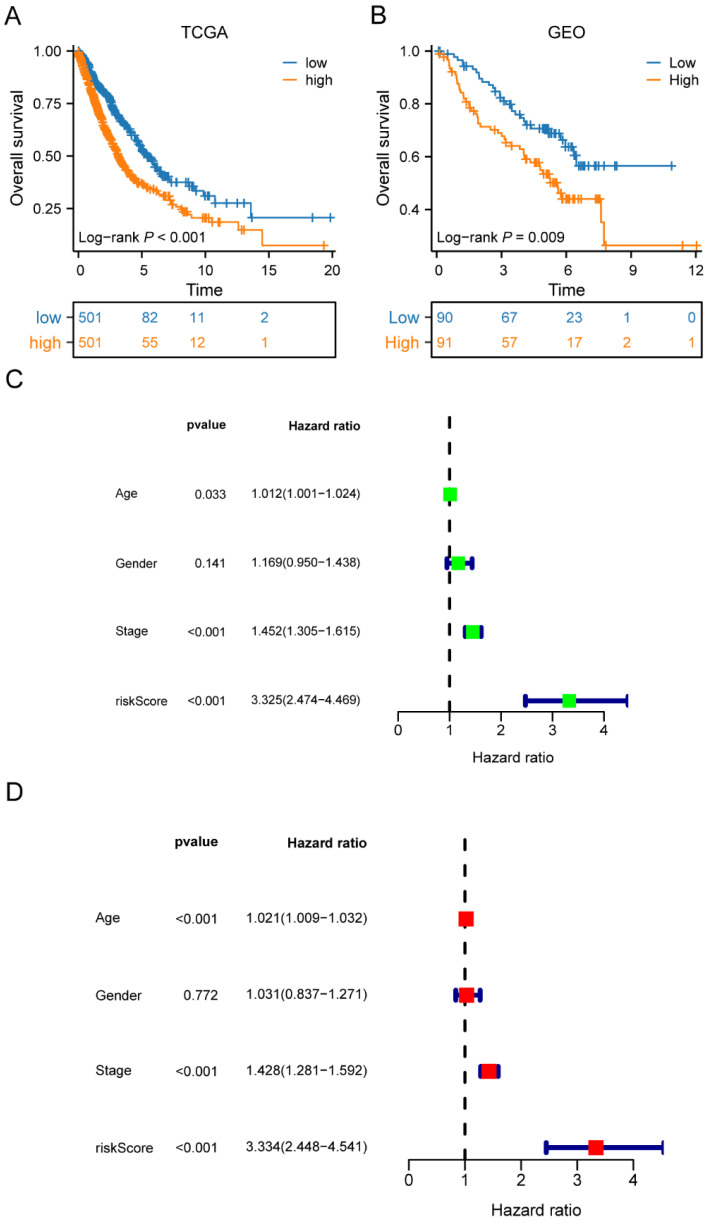
The risk score constructed by the 12 M2 macrophage-related genes in LC. (A) Survival curves of the high-risk and low-risk groups in TCGA cohorts; (B) Survival curves of high-risk and low-risk groups in GEO cohorts; (C-D) Univariate and multivariate analysis of the high-risk and low-risk groups in TCGA cohorts. LC, lung cancer; TCGA, the Cancer Genome Atlas; GEO, Gene Expression Omnibus.

**Figure 6:**
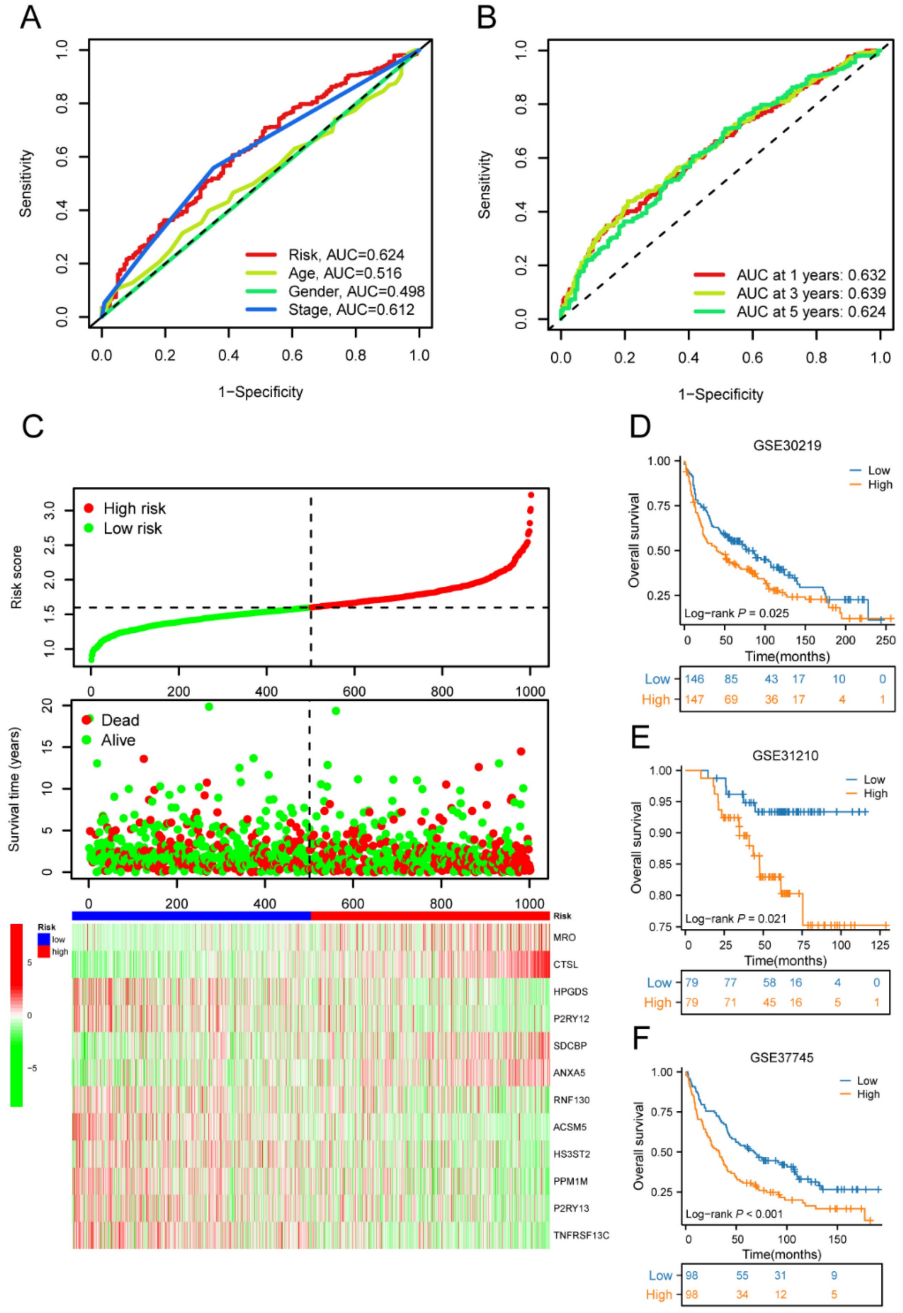
The ROC curve, risk diagram, and survival status distributions of the risk score. (A)The ROC curve of the risk score, age, gender, and stage; (B) The ROC curve for OS of the risk score at 1-, 3-, and 5-years; (C) The risk diagram and survival status distributions of the risk score; (D) Survival curves of high-risk and low-risk groups in GSE30219 cohorts; (E) Survival curves of high-risk and low-risk groups in GSE31210 cohorts; (F) Survival curves of high-risk and low-risk groups in GSE37745 cohorts. ROC, receiver operating characteristic; OS, overall survival.

Univariate Cox regression analysis was performed to screen 245 M2 macrophage-related genes. A total of 13 genes showed statistical significance in relation to overall survival (p<0.05) (**Figure 4A**). Following the application of LASSO regression analysis to reduce the scope of OS-related genes, a total of twelve genes were selected for the creation of the prognostic model (**Figure 4B-C**). The 12 genes included MRO, CTSL, HPGDS, P2RY12, SDCBP, ANXA5, RNF130, ACSM5, HS3ST2, PPM1M, P2RY13, and TNFRSF13C. Afterwards, the risk score associated with M2 macrophages was computed by multiplying the weights of these genes with their respective expression levels. The coefficient of these genes was shown in **Table 2**. In order to categorize patients into high-risk and low-risk groups, risk scores were computed for every individual in the TCGA and GSE50081 cohorts. Kaplan-Meier survival analysis demonstrated a significant association between OS and risk score, indicating that patients with a low-risk score had superior OS compared to those with a high-risk score (p<0.001) (**Figure 5A**). Then, using a separate cohort (GSE50081), we conducted a survival analysis to evaluate the validity of risk score. Notably, the results were comparable to those of the TCGA cohort (p<0.01) (**Figure 5B**). In addition, both univariate and multivariate Cox regression analyses showed that the risk score was an independent prognostic factor in LC (**Figure 5C-D**). The ROC curve showed that the area under the curve for the risk score is greater than that for age, gender, and stage (**Figure 6A**). ROC curves were also used to validate the risk score, which identified an area under the ROC curve for prediction of 1-, 3-, and 5-year OS of 0.632, 0.639, and 0.624, respectively (**Figure 6B**). The relationship between the 12 risk genes and survival was displayed in** Figure 6C** using a heat map.

**Figure 7:**
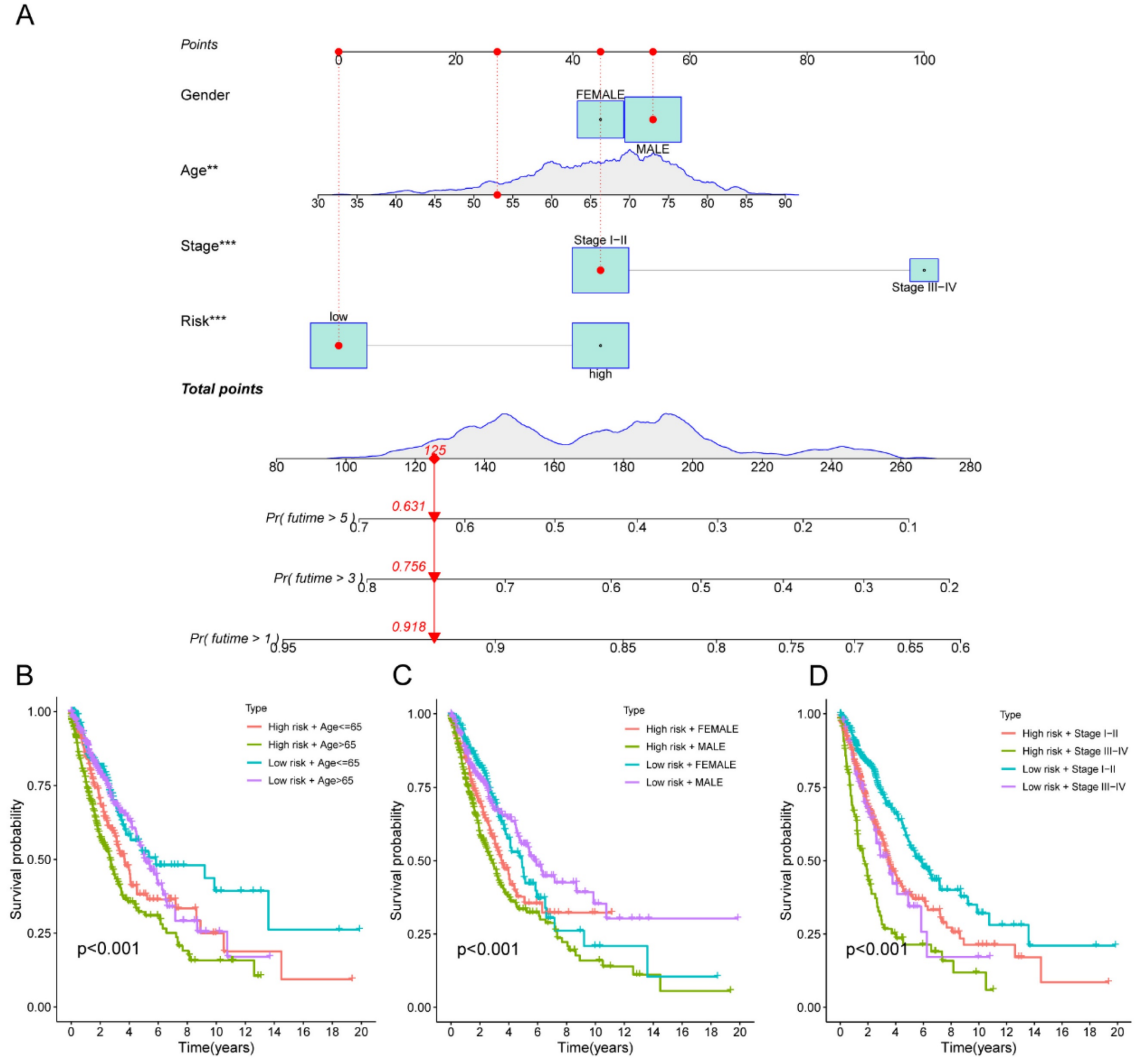
The macrophage-related model and subgroup prognostic analysis of the risk score. (A) The predicted model of OS for LC patients at 1-, 3-, and 5-years; (B) The subgroup prognostic analysis of risk score according to age; (C) The subgroup prognostic analysis of risk score according to gender; (D) The subgroup prognostic analysis of risk score according to stage. OS, overall survival; LC, lung cancer.

Moreover, the three GEO databases also validated that the risk score had a significant impact on the prognosis of LC patients, with high-risk group patients exhibiting significantly poorer outcomes compared to the low-risk group (all p<0.05) (**Figure 6D-F**). We then created a nomogram to predict the 1-, 3-, and 5-year survival of patients in order to improve the use of the risk score in the clinic. This was accomplished by integrating the risk score with additional clinicopathological indicators. The predicted probabilities of OS for LC patients at 1-, 3-, and 5-years were 0.918, 0.756, and 0.631, respectively. (**Figure 7A**). Finally, we conducted a prognostic analysis of high- and low-risk patients in different subgroups based on age, gender, and stage. The results showed that the prognosis of high-risk group was worse than that of low-risk group (p<0.001) (**Figure 7B-C**).

### Prognostic value of risk score in LC progression

**Figure 8:**
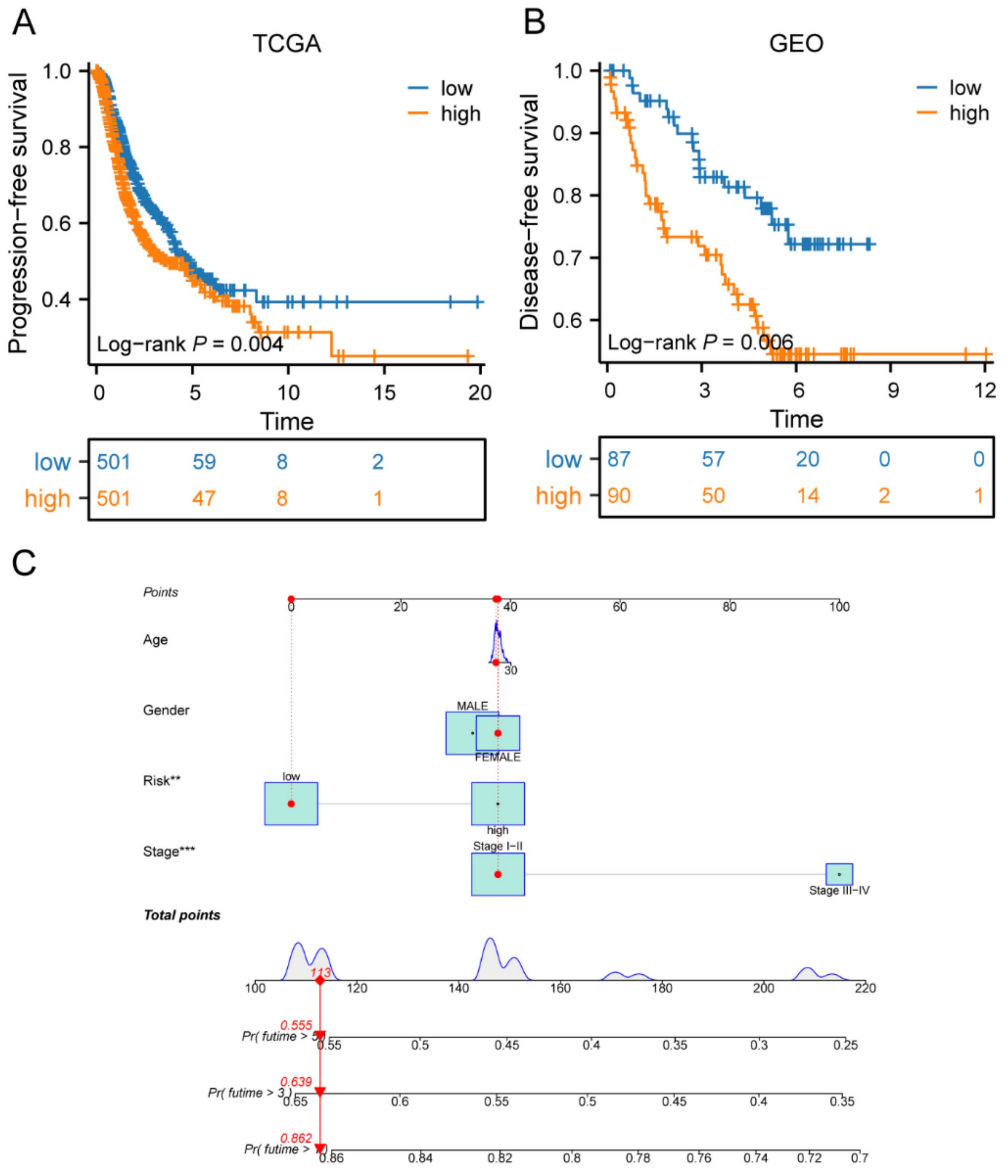
Prognostic value of risk score in LC progression. (A) Survival curves of the high-risk and low-risk groups in TCGA cohorts; (B) Survival curves of high-risk and low-risk groups in GEO cohorts; (C) The predicted model of progression-free survival for LC patients at 1-, 3-, and 5-years. LC, lung cancer; TCGA, the Cancer Genome Atlas; GEO, Gene Expression Omnibus.

To further investigate the impact of the risk score on patient prognosis, we also conducted a prognostic analysis of progression-free survival and disease-free survival in high- and low-risk groups. In the TCGA cohort, our results showed that patients in the high-risk group had significantly lower progression-free survival rates compared to those in the low-risk group (p<0.01) (**Figure 8A**). Additionally, in the validation cohort from GEO, we found that patients in the high-risk group also had poorer disease-free survival rates than those in the low-risk group (p<0.01) (**Figure 8B**). Finally, we combined the risk score with other clinical factors to construct a model for predicting PFS in LC patients for 1-, 3-, and 5-years. The predicted probabilities of PFS for LC patients at 1-, 3-, and 5-years were 0.862, 0.639, and 0.555, respectively (**Figure 8C**).

### GSEA of high- and low-risk groups

**Figure 9:**
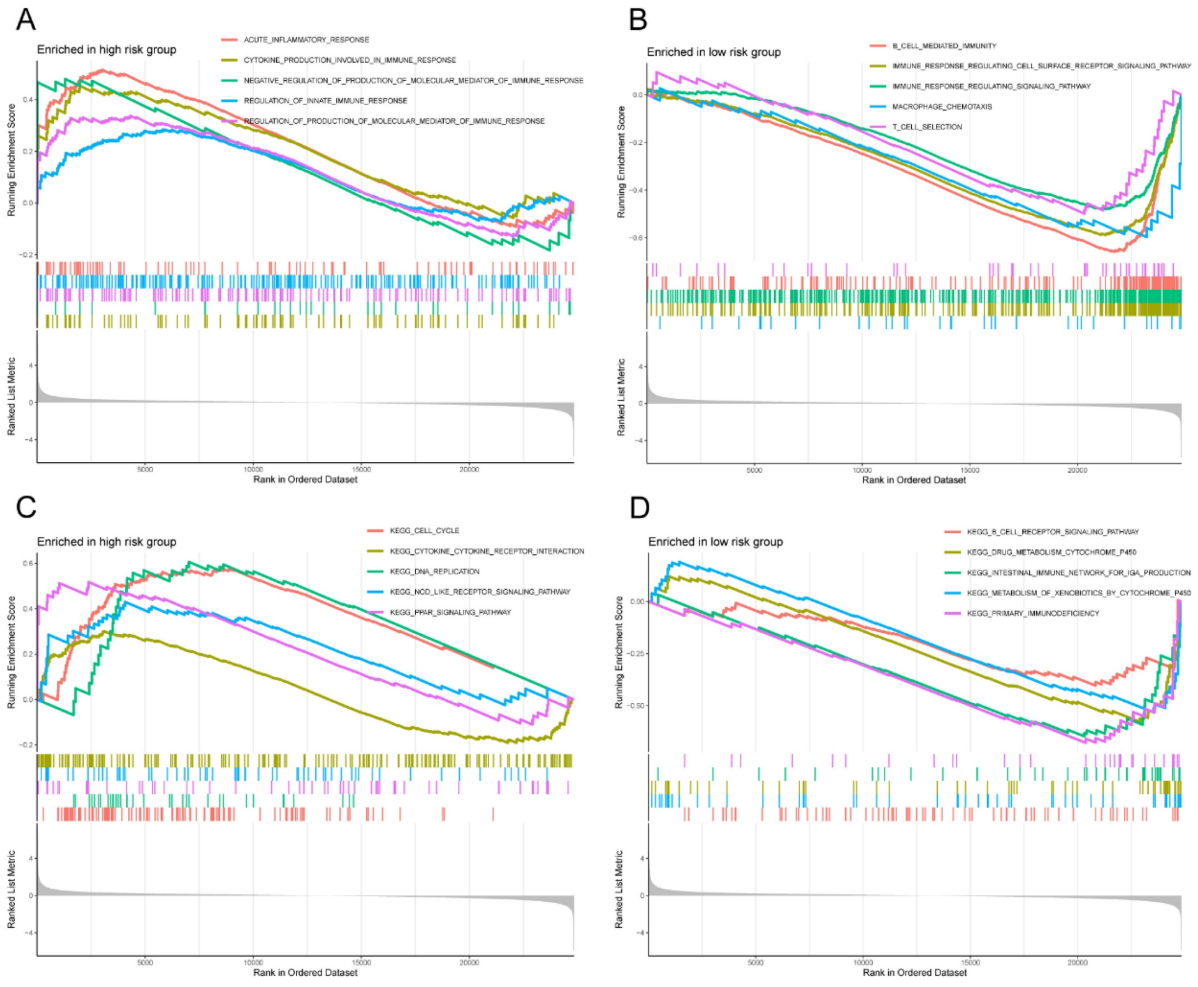
GSEA of high- and low-risk groups. (A) The significantly signature functions enriched in the high-risk group; (B) The significantly signature functions enriched in the low-risk group; (C) The significantly signature pathways enriched in the high-risk group; (D) The significantly signature pathways enriched in the low-risk group. GSEA, Gene set enrichment analysis.

In light of the correlation between the 12 M2 macrophage-related genes and prognosis, we conducted a more detailed examination of the signature functions and pathways associated with the low-risk and high-risk groups by utilizing GSEA. The signature functions of the high-risk group were enriched in inflammatory response, cytokine production involved in immune response, negative regulation of production of molecular mediator of immune response, regulation of innate immune response, and regulation of production of molecular mediator of immune response (**Figure 9A**), whereas the signature functions of the low-risk group were enriched in the B cell mediated immunity, immune response regulating cell surface receptor signaling pathway, immune response regulating signaling pathway, macrophage chemotaxis, and T cell selection (**Figure 9B**). Besides, the significantly signature pathways enriched in the high-risk group were cell cycle, cytokine cytokine receptor interaction, DNA replication, nod like receptor signaling pathway, and PPAR signaling pathway (**Figure 9C**). Finally, the significantly signature pathways enriched in the low-risk group were B cell receptor signaling pathway, drug metabolism cytochrome, intestinal immune network for IgA production, metabolism of xenobiotics by cytochrome P450, and primary immunodeficiency (**Figure 9D**). These results indicated that the risk score was closely associated with the immune and metabolic status of the LC patients.

### Immune characteristics of high- and low-risk groups

**Figure 10:**
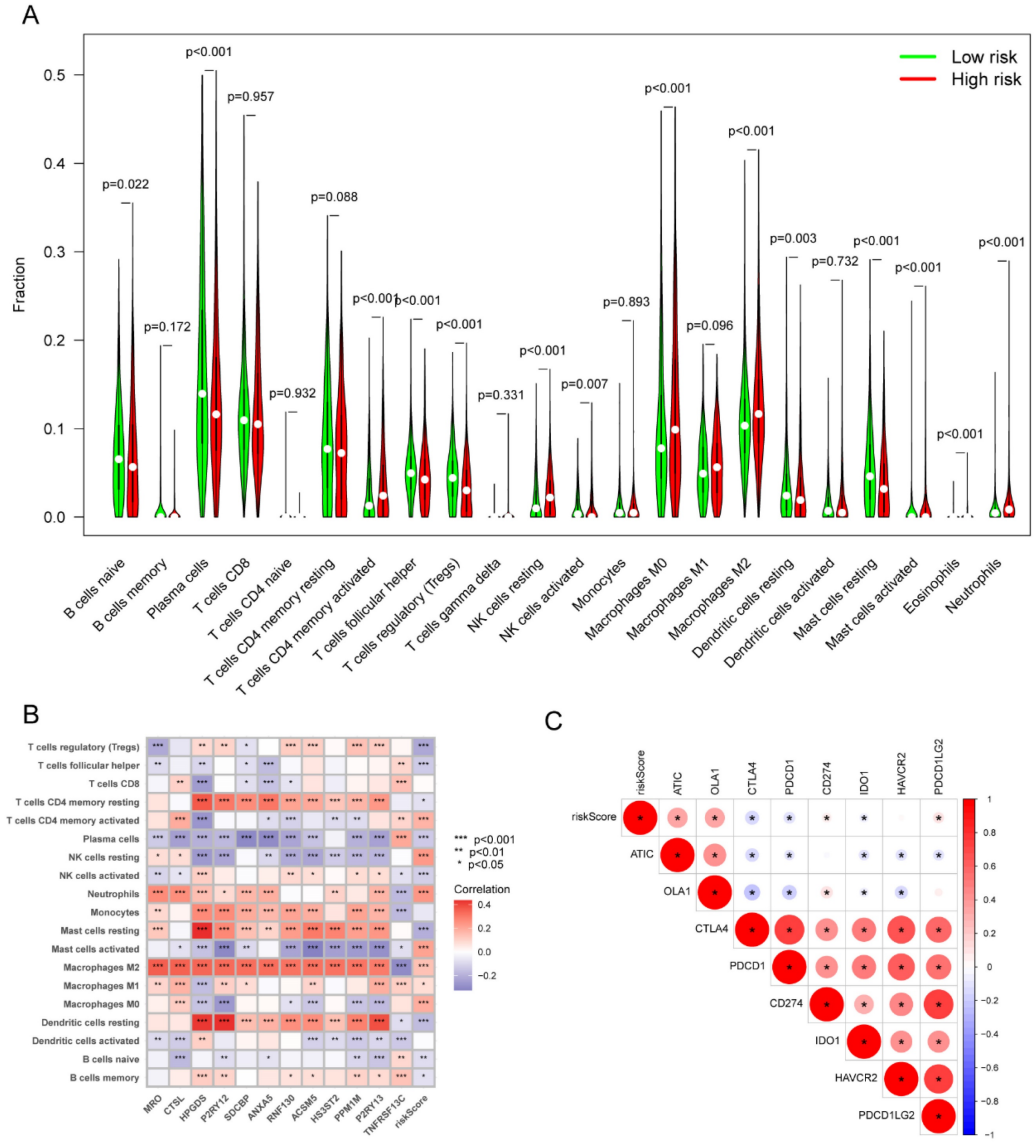
The correlation of the risk score with immune infiltration in the TCGA cohort. (A) The correlation between different risk groups and immune cell infiltration levels in LC patients (CIBERSORT algorithm); (B) The correlation between the 12 risk genes and the risk score with immune cells infiltration in LC patients; (C) The correlation between the risk score and immune checkpoint-related genes in LC patients. TCGA, the Cancer Genome Atlas; LC, lung cancer.

**Figure 11:**
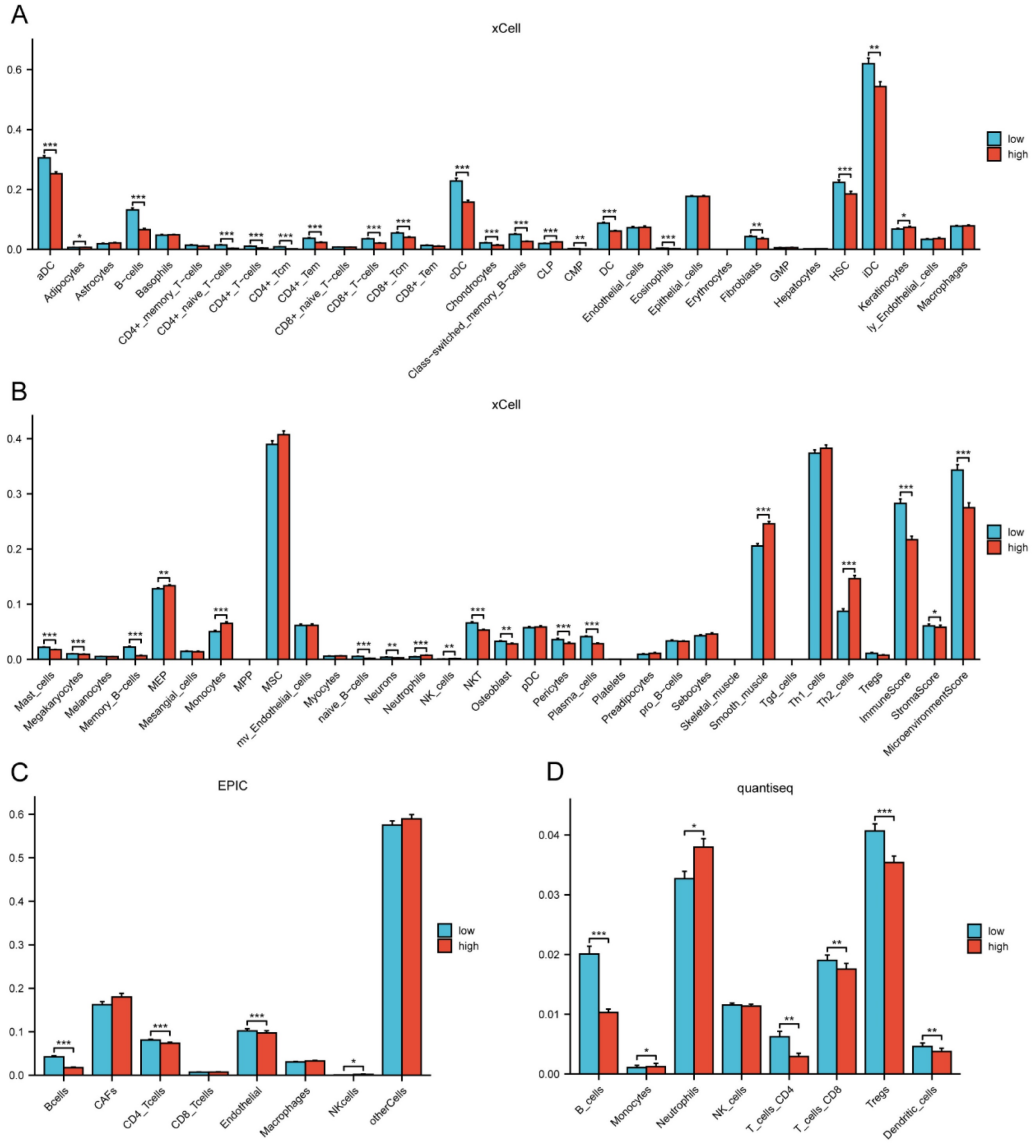
The correlation of the risk groups with immune cell infiltration in the TCGA cohort. (A-B) The correlation between different risk groups and immune cell infiltration levels in LC patients (xCell algorithm); (C) The correlation between different risk groups and immune cell infiltration levels in LC patients (EPIC algorithm); (D) The correlation between different risk groups and immune cell infiltration levels in LC patients (quantiseq algorithm). TCGA, the Cancer Genome Atlas; LC, lung cancer.

**Figure 12:**
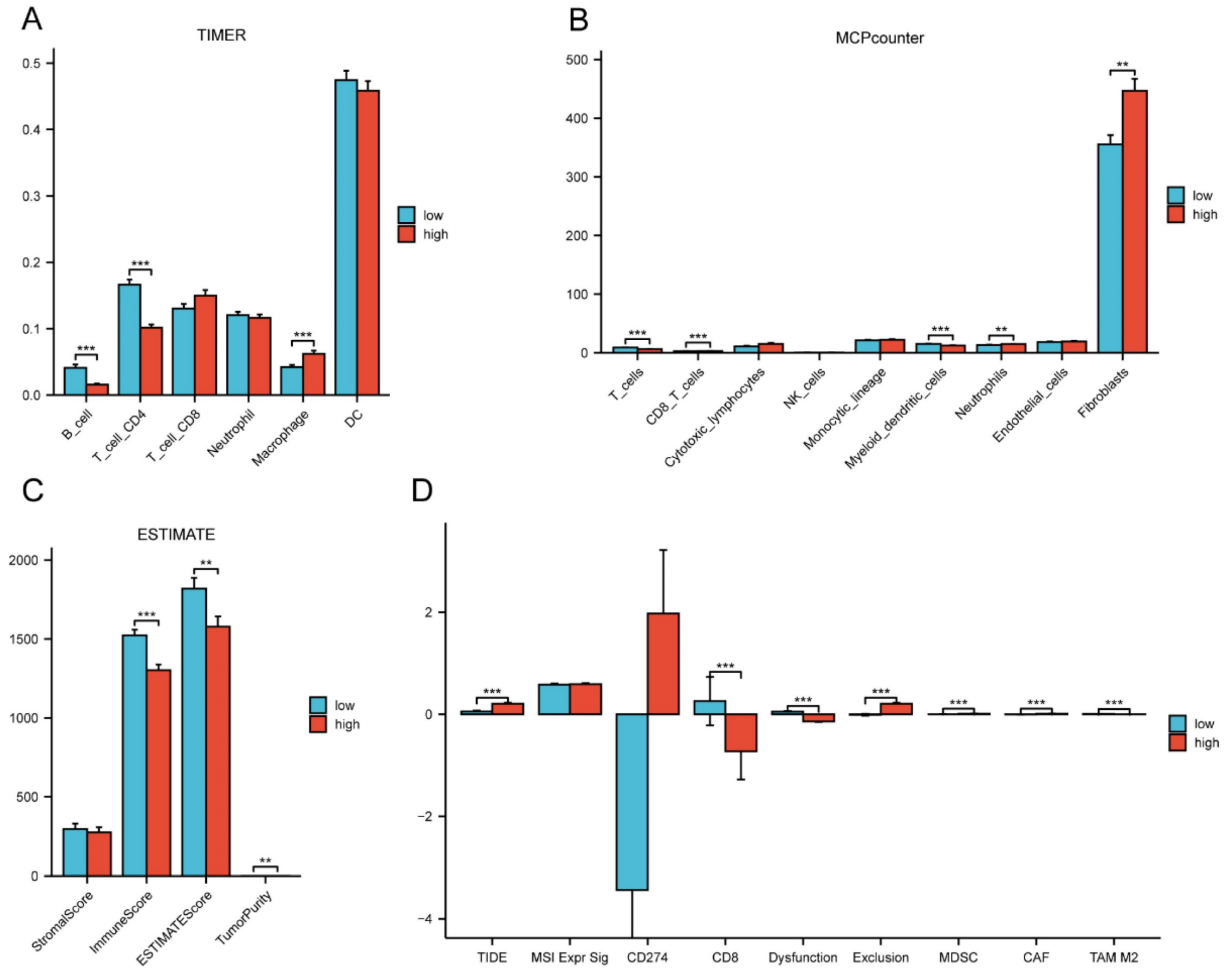
The correlation of the risk groups with immune cell infiltration and immunotherapy response in the TCGA cohort. (A) The correlation between different risk groups and immune cell infiltration levels in LC patients (TIMER algorithm); (B) The correlation between different risk groups and immune cell infiltration levels in LC patients (MCPcounter algorithm); (C) The correlation between different risk groups and immune cell infiltration levels in LC patients (ESTIMATE algorithm); (D) The differences of immunotherapy response between different risk groups in LC patients. TCGA, the Cancer Genome Atlas; LC, lung cancer.

By comparing the distribution of 22 different types of immune cells in different risk groups, the CIBERSORT algorithm was used to investigate the immune cell composition in the two groups. Our results demonstrated that B cell naïve, plasma cells, dendritic cells resting, and mast cells resting were more abundant in the low-risk group whereas macrophages M0, macrophages M2, natural killer cells resting, and neutrophils were more plentiful in the high-risk group (p<0.05) (**Figure 10A**). The visualization in **Figure 10B** illustrated the correlation between the 12 risk genes and the risk score with immune cells infiltration. A higher prevalence of macrophages M2 infiltration was linked to a lower survival rate. We also explored the correlation between the risk score and immune checkpoint-related genes. We found that the risk score of LC patients was negatively correlated with PD-1 and CTLA-4 (p<0.05) (**Figure 10C**). This suggested that patients in the high-risk group might have poorer immune responses. To further explore the relationship between the risk score and immune cell infiltration, we applied another six algorithms for validation. **Figure 11A-D** respectively demonstrated the relationship between high- and low-risk groups and immune cell infiltration using the xCell, EPIC, and quantiseq algorithms. **Figure 12A-C** then respectively used TIMER, MCPcounter, and ESTIMATE algorithms to validate the relationship between high- and low-risk groups and immune cell infiltration. Interestingly, these results also indicated that patients in the high-risk group were associated with lower infiltration of B cell, CD8+ cell, and CD4+ cell (p<0.05). This was consistent with our previous results and further suggested a potential association between high-risk patients and poorer immunotherapy response.

### Analysis of immunotherapy prediction in high- and low-risk groups

TIDE was used to evaluate the differences in immunotherapy sensitivity between the two groups. It was discovered that CD8, T cell dysfunction, and T cell exclusion showed variation between the two risk groups. Additionally, the TIDE score was higher in the high-risk group (p<0.001) (**Figure 12D**). A greater TIDE prediction score was associated with a higher possibility of immune evasion, indicating that these patients were less likely to benefit from immunotherapy. In addition, Myeloid Derived Suppressor Cell (MDSC) was also shown to be higher in the high-risk group (p<0.001). The elevated exclusion score of the high-risk group further indicated a greater potential for immunological evasion (p<0.001). Collectively, these findings suggested that the risk score significantly influenced the tumor immune microenvironment.

### Drug sensitivity analysis of high- and low-risk groups

**Figure 13:**
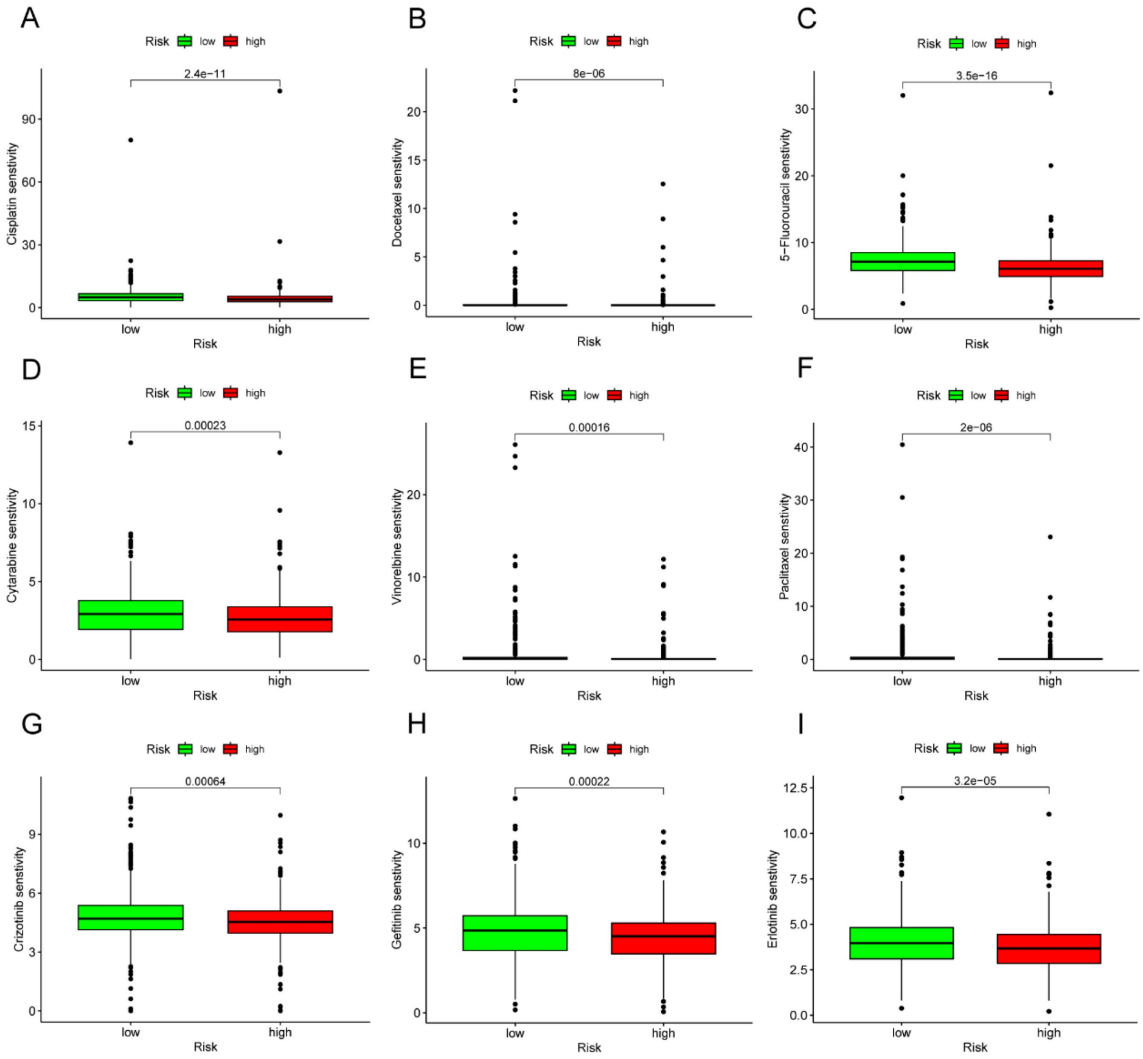
Drug-sensitivity analysis based on different risk group in LC. (A) Analysis of drug sensitivity for chemotherapeutic drug in high- and low-risk group. (B) Analysis of drug sensitivity for targeted drug in high- and low-risk group. LC, lung cancer.

In the TCGA cohort, we sought to identify associations between different risk groups and the efficacy of therapy for treating LC patients. The results revealed that the high-risk group was associated with a lower IC50 for chemo-therapeutics such as Cisplatin, Docetaxel, 5-Fluorouracil, Cytarabine, Vinorelbine, and Paclitaxel (p<0.001) (**Figure 13A-F**). In addition, the high-risk group was associated with a low IC50 in targeted drug, including Crizotinib, Gefitinib, and Erlotinib (p<0.001) (**Figure 13G-I**).

### Characterization of RNF130 expression in several datasets and *in vitro* assays

**Figure 14:**
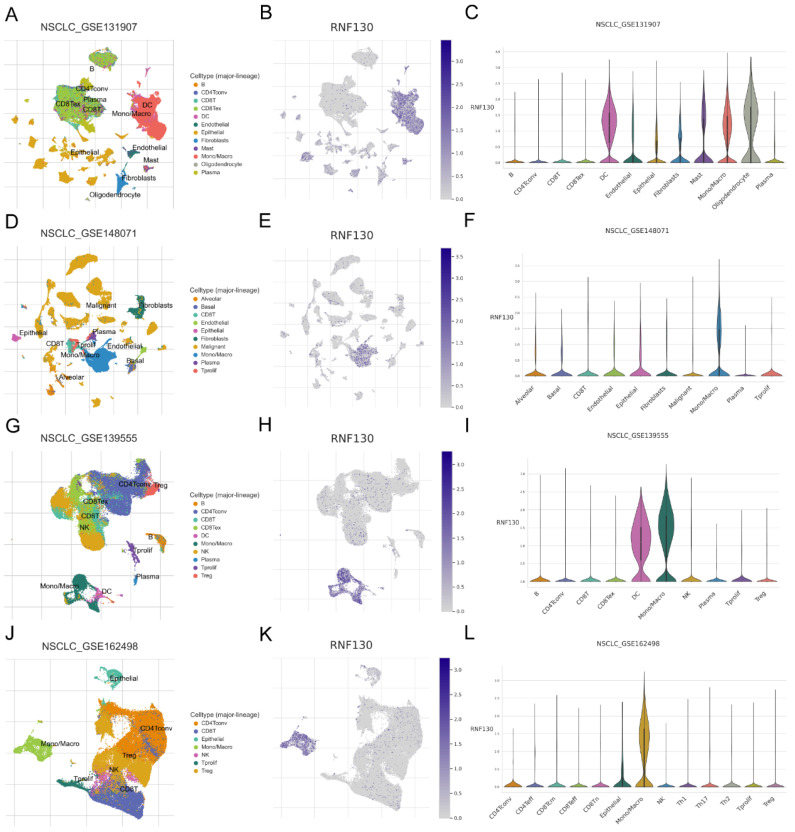
Analysis of RNF130 expression in several cell types at the single cell level. Single-cell mapping for visualizing RNF130 expression levels in different cell types in the NSCLC_GSE131907(A-C), NSCLC_GSE148071(D-F), NSCLC_GSE139555(G-I), and NSCLC_GSE163498(J-L) datasets.

**Figure 15:**
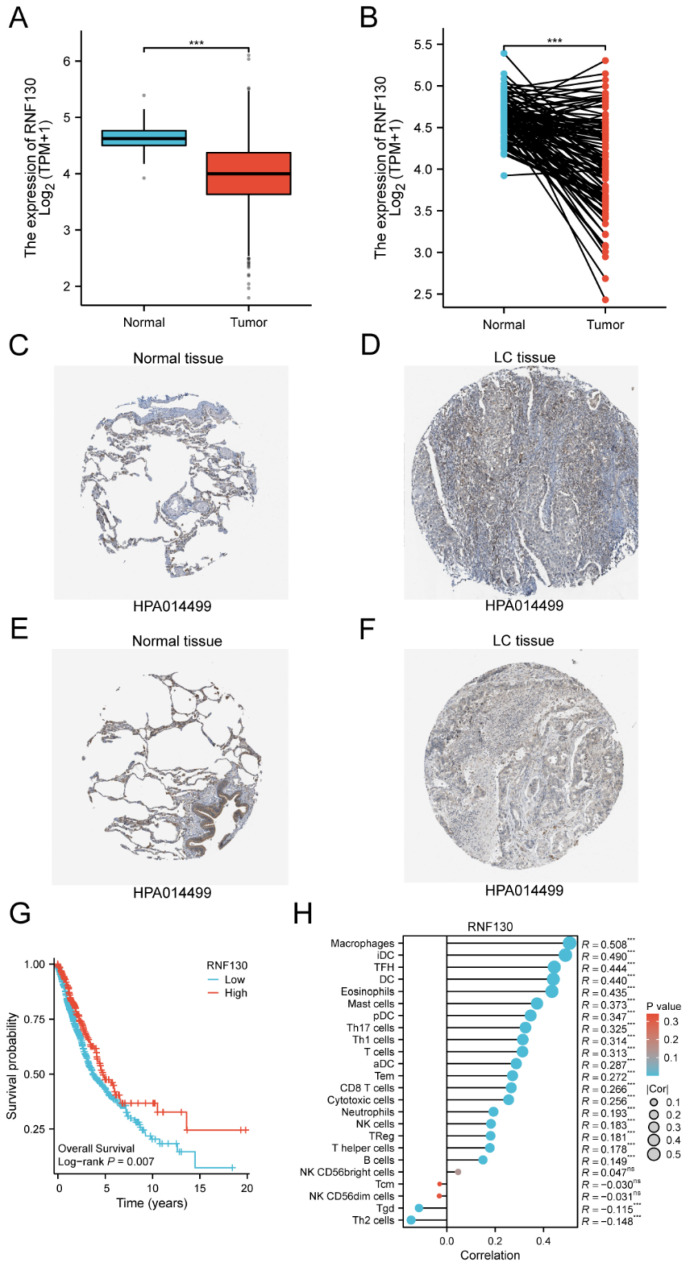
The expression levels of RNF130 in LC. (A) Expression levels of RNF130 in non-paired tumor and normal samples; (B) Expression levels of RNF130 in paired tumor and normal samples. The expression levels of RNF130 in LC were determined using the HPA. (C) Medium; (D) Not detected; (E) Medium, and (F) Low; (G)The overall survival between high RNF130 and low RNF130 expression groups; (H)The correlation between RNF130 expression and the infiltration of immune cells. LC, lung cancer; HPA, Human Protein Atlas.

**Figure 16:**
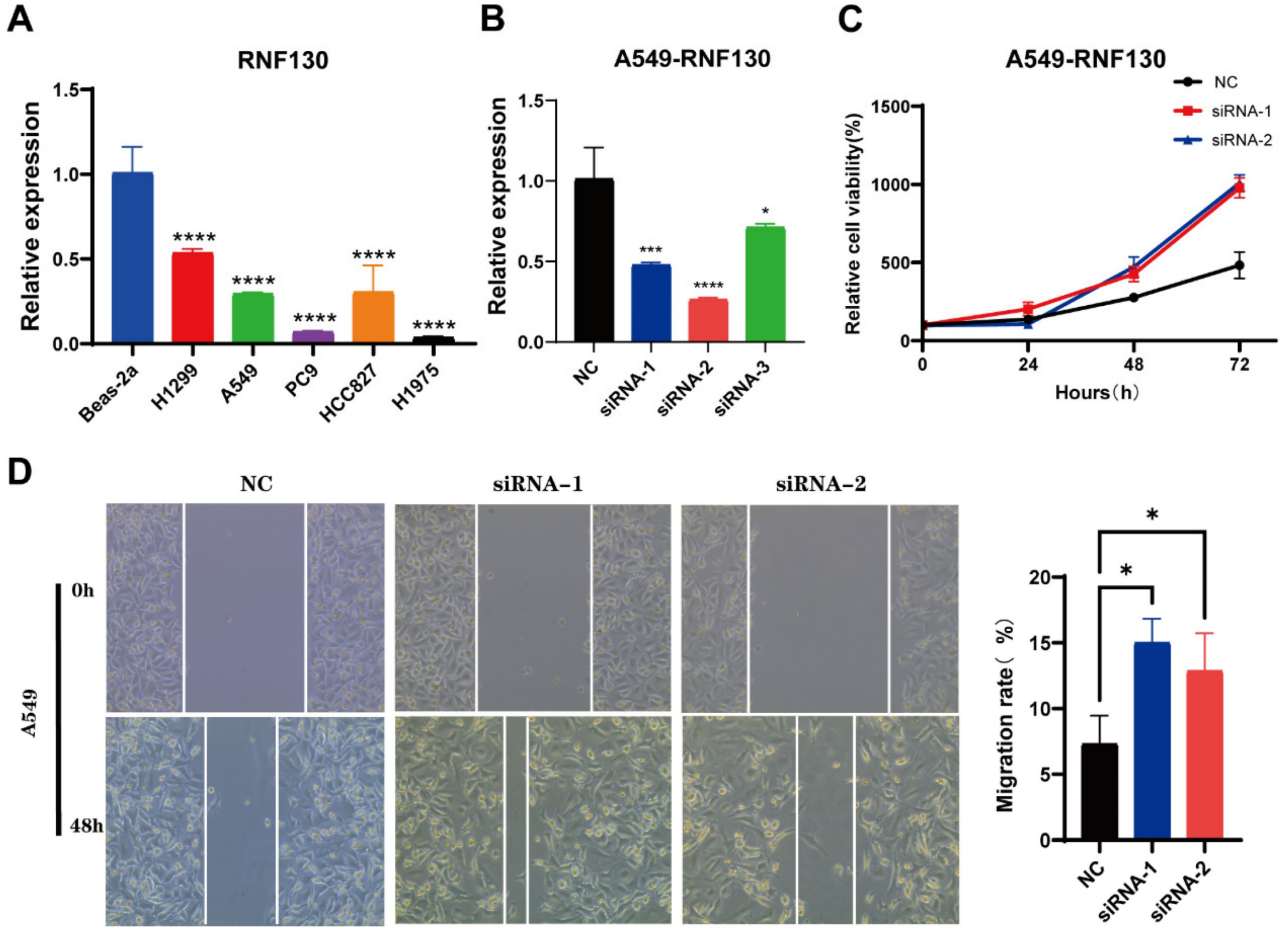
The characterization of RNF130 expression and *in vitro* assays in LC. (A) The expression of RNF130 in LC cells and normal bronchial epithelial cell line (Beas-2a); (B) The knockdown efficiency of siRNA-1, siRNA-2, and siRNA-3. (C) CCK8 assay of NC, siRNA-1, and siRNA-2 group; (D) The wound healing assay of NC, siRNA-1, and siRNA-2 group. LC, lung cancer; NC, normal control.

We examined the RNF130 expression in LC using single-cell datasets (NSCLC_GSE131907, NSCLC_GSE148071, NSCLC_GSE139555, and NSCLC_GSE163498) from the TISCH database. The data indicated that macrophages were the primary site of RNF130 distribution (**Figure 14A-L**). The data suggested that RNF130 was associated with M2 macrophage polarization. We also discovered that LC samples from the TCGA database exhibited significantly lower levels of RNF130 mRNA expression than normal tissues (p<0.001) (**Figure 15A-B**). The results from the HPA database further demonstrated a trend toward a low expression of RNF130 at the protein level in LC (**Figure 15C-F**). Compared to low RNF130 mRNA expression, the prognosis for high RNF130 mRNA expression level was favorable (p<0.01) (**Figure 15G**). Importantly, we discovered that a significant percentage of immune cells had positive correlations with RNF130 expression (p<0.05) (**Figure 15H**). Furthermore, we assessed the RNF130 expression level in five distinct lung cancer cell lines and a normal bronchial epithelial cell line. Similarly, our results showed that the expression of RNF130 was lower the five types of LC cells (p<0.001) (**Figure 16A**). To better investigate the role of RNF130 in LC, we transfected A549 cells with RNF130 siRNA, and used qRT-PCR to assess the efficacy of RNF130 transfection. Given that the qRT-PCR results indicated siRNA-1 and siRNA-2 had the most effective knockdown, we selected these two siRNAs for subsequent experiments (p<0.001) (**Figure 16B**). The CCK8 assay showed that the proliferation capacity of A549 cells after siRNA treatment was significantly higher than that of the normal control (NC) group (p<0.001) (**Figure 16C**). Finally, the wound healing assay demonstrated that the migration ability of A549 cells after siRNA treatment was significantly greater than that of the NC group (p<0.05) (**Figure 16D**). These results suggested that suppressing RNF130 can promote the proliferation and migration of LC.

## Discussion

Notwithstanding the advancements that has been made in screening and treatment, LC continues to be the most common type of cancer and the primary cause of death from cancer in the world 1. The interaction between the tumor immune microenvironment and genetic alterations is a key component in the intricate and ever-changing process of LC occurrence and progression 30. Whether for patients with lung adenocarcinoma or lung squamous cell carcinoma, immunotherapy is one of the most prominent treatment modalities among many. It has changed the landscape of anti-tumor therapy and ushered in a new era of anti-tumor treatment 3. While immune checkpoint inhibitors have demonstrated significant effectiveness in the treatment of LC, only a small proportion of patients have a positive response to these therapies 31. Hence, there remains a need for further clarification regarding the screening of groups that would benefit from immunotherapy and the predictors associated with this benefit.

Macrophages, which are intrinsically malleable immune cells, undergo activation through the integration of microenvironmental signals 5, 9, 32. More and more evidence pointed to the important roles played by tumor-associated macrophages as mediators between the immune system's potential antitumor effector mechanisms and the tumors' anti-immune defenses 5, 33. Given the important role macrophages in tumor immunity and their close relationship with LC, this could provide a breakthrough in fully examining the immunological landscape in LC. Nevertheless, limited research has naturally concentrated on the characteristics of the tumor immune microenvironment and prognostic prediction from the perspective of macrophage-related genes in LC. Consequently, there is an urgent need to investigate the immunological subtypes of LC.

In the present study, we developed a risk score incorporating 12 identified M2 macrophage-related genes, specifically focusing on LC patients with TCGA cohort. We then verified the dependability of this risk score by testing it on GEO cohort. Patients were divided into high- and low- risk groups according to the risk score. The risk score stood out as an independent prognostic factor for LC patient prognosis, according to our findings. When contrasted with the low-risk group, the high-risk patients' prognosis was substantially poorer. A statistically significant correlation between risk score and immunity was found in the GSEA. In addition, we used seven immunological algorithms to investigate immune cell infiltration in various risk groups. Immune cell infiltration of B cells, CD4+ cells, and CD8+ cells was negatively correlated with high-risk group patients. Patients in the high-risk group are more prone to immune evasion and might experience worse immunotherapy outcomes, as we further confirmed by utilizing an immunotherapy response database. Ultimately, by doing drug sensitivity study, we discovered that individuals classified in the high-risk group exhibited greater sensitivity to several chemotherapeutic and targeted medications compared to those in the low-risk group. At the single-cell level, RNF130 expression was found to be substantially concentrated in macrophages. Moreover, RNF130 had low expression levels in LC tumor tissues and was found to be correlated with prognosis. We investigated RNF130 expression in LC cells and found that it was similarly underexpressed in these cells, Finally, *in vitro* experiments suggested that suppressing RNF130 can promote the proliferation and migration of LC.

The occurrence and development of cancer result from the interaction of multiple genes and signaling pathways. Merely concentrating on a small number of genetic indicators is inadequate for establishing a correlation between LC immunological response and prognosis. Additionally, discovering potential biomarkers and therapeutic targets through the exhaustive and methodical profiling of different immune cells from diverse tumor samples, relying solely on experimental evidence, is a challenging and time-consuming process. Through the use of advanced computational methods, bioinformatics techniques are able to directly extract information relevant to cell types 34, 35. In light of this, we developed a risk score model and a subgroup categorization system for 12 M2 macrophage-related genes. Eight of these genes were considered to be favorable prognostic genes in individuals with LC, whereas the remaining four genes were considered to be adverse prognostic genes. The bioinformatics methods were used in our study to investigate the immune microenvironment and M2 macrophage-related genes in different LC patients. The risk score demonstrated good performance on external and independent datasets. Consequently, we deduced that the risk score possessed both a high level of clinical feasibility and applicability.

Due to the high concentration of T cells in healthy lung tissue, LC serves as a model disease for investigating cancer immunosurveillance 36. In addition, LC shows promising reactions to immune checkpoint inhibitors (ICIs) that target T cells through the PD-1/PD-L1 and CTLA-4 pathways 37-39. Nevertheless, only a small proportion of patients can derive benefits from ICIs treatment. Our research results suggested that patients with higher risk scores had lower levels of infiltration by major immune cells (B cells, CD4+ cells, and CD8+ cells) and were significantly negatively correlated with ICIs (PD-1 and CTLA4). Another important finding was that patients with higher risk scores were more likely to experience immune escape and might have a poorer response to immunotherapy. The effectiveness of the antitumor immune response is a key factor in determining the prognosis of numerous solid tumors. Monoclonal antibodies targeting PD-1/PD-L1 and CTLA4 have greatly enhanced the survival outlook for individuals with cancer. Furthermore, several studies have illustrated that the survival rate of cancer patients is closely related to the degree of infiltration of CD8+ T cells within the tumor 40. Therefore, our risk score might have the potential to offer significant insights into the prediction of immunotherapy response and prognosis, as well as the direction of clinical practice.

We further explored the sensitivity of chemotherapeutic and targeted drugs in LC patients across the two risk groups. The results indicated that patients in the high-risk group were more sensitive to several commonly used chemotherapeutic and targeted drugs in LC patients. Following the application of the predictive risk score, these drugs were identified as potential treatments for LC in certain circumstances. Our results demonstrated a promising potential for guiding individualized strategies and managing chemo/targeted therapy. Nonetheless, additional research is required to investigate the relationship between the risk score and the underlying biological mechanisms.

The risk score provides a personalized prognostic tool that can identify high-risk patients who may benefit from more aggressive treatment strategies or closer monitoring. By predicting immune cell infiltration and potential responses to immunotherapy, the risk score can guide clinicians in selecting appropriate immunotherapeutic approaches for LC patients. Besides, the identification of high-risk patients who are more sensitive to certain chemotherapeutic and targeted drugs enables the development of tailored treatment regimens, potentially improving patient outcomes. Finally, the significant associations between the risk score and immune-related functions open new avenues for research into the underlying biological mechanisms, potentially leading to the discovery of novel therapeutic targets. Nevertheless, even though there have been some encouraging outcomes, there are still certain limitations. Firstly, public databases, such as the TCGA and GEO, served as the basis for the study. These databases are wonderful resource; nevertheless, they have limitations, including the possibility of heterogeneity in terms of data quality and patient characteristics. Secondly, our research demonstrated a substantial correlation between the prognosis of LC patients and the twelve M2 macrophage-related genes. This correlation was determined only through the process of data mining. In order to shed light on the function and mechanisms of these genes, additional experimental research to be conducted is required. Furthermore, the risk score was employed to simulate patient response to treatment with ICIs. However, the current number of immunotherapy cohorts is insufficient to fully verify our results, thus the risk score remains insufficient to entirely substitute the actual treatment response. Notwithstanding these constraints, the advancement of bioinformatics has undeniably aided researchers in discovering prospective therapeutic targets for LC. Despite these limitations, the progress of bioinformatics has undoubtedly benefited researchers in identifying potential therapeutic targets for LC. Hence, additional prospective studies are still required.

## Conclusion

In summary, we constructed a twelve-gene risk score and verified it using independent LC cohorts. The high-risk group constructed with M2 macrophage-related genes in LC was closely associated with poor prognosis, low immune cell infiltration, and poorer response to immunotherapy. These findings could serve as a theoretical foundation for future investigations and the development of precise, personalized immunotherapy for patients with LC. This risk score can help differentiate and predict the prognosis and immune status of LC patients.

## Supplementary Material

Supplementary tables.

## Figures and Tables

**Table 1:** The clinical characteristics of the LC patients.

Clinicopathologic variable		Total (N)	Percentage (%)
Gender			
	Male	599	59.8
	Female	403	40.2
Age			
	≤65 years old	423	42.2
	>65 years old	551	55.0
	Unknown	28	2.8
T stage			
	T1	283	28.2
	T2	559	55.8
	T3	116	11.6
	T4	41	4.1
	Tx	3	0.3
N stage			
	N0	641	64.0
	N1	224	22.4
	N2	112	11.2
	N3	7	0.7
	Nx	17	1.7
	Unknown	1	0.1
M stage			
	M0	749	74.8
	M1	31	3.1
	Mx	214	21.4
	Unknown	8	0.8
TNM stage			
	Stage I	511	51.0
	Stage II	283	28.2
	Stage III	164	16.4
	Stage IV	32	3.2
	Unknown	12	1.2
Risk group			
	Low	501	50.0
	High	501	50.0
Vital status			
	Dead	397	39.6
	Alive	605	60.4

**Table 2:** The coefficient of 12 M2 macrophage-related genes.

Gene	Coefficient
MRO	0.12539
CTSL	0.226438
HPGDS	-0.01027
P2RY12	-0.02269
SDCBP	0.106269
ANXA5	0.085241
RNF130	-0.12156
ACSM5	-0.0859
HS3ST2	-0.0563
PPM1M	-0.09421
P2RY13	-0.06242
TNFRSF13C	-0.02262

## Abbreviations

LC: lung cancer
OS: overall survival
TCGA: The Cancer Genome Atlas
GEO: Gene Expression Omnibus
LASSO: least absolute shrinkage and selection operator
GSEA: gene set enrichment analysis
HPA: Human Protein Atlas
qRT‑PCR: quantitative real‑time polymerase chain reaction
GO: gene ontology
KEGG: Kyoto Encyclopedia of Genes and Genomes
ROC: receiver operating characteristic
NES: normalized enrichment score
FDR: false discovery rate
TIDE: tumor immune dysfunction and exclusion
GDSC: genomics of drug sensitivity in cancer
TISCH: Tumor Immune Single Cell Center
IC50: half inhibitory concentration
BP: biological processes
CC: cellular components
MF: molecular function
MDSC: Myeloid Derived Suppressor Cell
ICIs: immune checkpoint inhibitors
