# Supplementary material for: Prognostic and immunotherapeutic significances of M2 macrophage-related genes signature in lung cancer
Source: J Cancer. 2024 Jul 22;15(15):4985–5006. doi: 10.7150/jca.98044 (PMC11310876; doi:10.7150/jca.98044)
Supplement: Supplementary file 1 — Supplementary tables. [file jcav15p4985s1.pdf]

Supplementary Table s1 The primer sequences involved in this study.

Primer	5' to 3'
Actin-F	GAGAAAATCTGGCACCACACC
Actin-R	GGATAGCACAGCCTGGATAGCAA
RNF130-F	TCAACATTGCAGTAACAAGTGG
RNF130-R	TACATGTCCAAAGAAGGTTCGA

Supplementary Table s2 The siRNA sequence of RNF130.

Genes	Top strand	Bottom strand
siRNF130-1	CAGAAGAUCAGGUACACAATT	UUGUGUACCUGAUCUUCUGTT
siRNF130-2	CUGCAUAGAGAGCUAUAAGTT	CUUAUAGCUCUCUAUGCAGTT
siRNF130-3	GUGCCGAAUUUGCCAUGUATT	UACAUGGCAAAUUCGGCACTT
